# Impacts of prolonged chlorpyrifos exposure on locomotion and slow-and fast- twitch skeletal muscles contractility in rats

**DOI:** 10.1016/j.toxrep.2019.06.006

**Published:** 2019-06-13

**Authors:** Nancy Hallal, Hiba El Khayat El Sabbouri, Ali Salami, Wiam Ramadan, Hassan Khachfe, Mohamed E. Moustafa, Mahmoud Khalil, Wissam H. Joumaa

**Affiliations:** a Department of Biological Sciences, Faculty of Science, Beirut Arab University, Lebanon; b Laboratoire Rammal Hassan Rammal, Equipe de recherche PhyToxE, Faculté des Sciences (section V), Université libanaise, Nabatieh, Lebanon; c Department of Biochemistry, Faculty of Sciences, Alexandria University, Egypt; d Lebanese Institute for Biomedical Research and Application (LIBRA), International University of Beirut (BIU), Beirut, Lebanon; e PERITOX UMR-I-0, University of Picardie Jules Verne, 80025, Amiens, France

**Keywords:** Chlorpyrifos, Contraction, *Extensor digitorum longus*, Locomotion, Soleus

## Abstract

**Aim:**

Investigate the effect of dietary exposure to chlorpyrifos on locomotion and contraction of soleus and*extensor digitorum longus* (*edl*) involved in locomotion. Methods: Rats were fed diets containing 1 or 5 mg kg^−1^ of chlorpyrifos for six weeks. Locomotion has been assessed weekly using beam walking and beam balance tests. Soleus and *edl* were removed to study contractile properties, myofibrillar protein content and myosin heavy chain isoforms.

**Results:**

Animals treated with 5 mg kg^−1^ chlorpyrifos had a decrease body weight. An increase by 28% and 24% in latency time assessed by beam walking test and a decrease by 9% and 13% in the beam balance time was reported after 6 weeks of 1 and 5 chlorpyrifos exposure respectively. The contractile properties in soleus showed an increase in twitch amplitude by 25% and 63% in 1 and 5 doses respectively, without modification in the contraction time and half relaxation time. *edl* treated with 1 mg kg^−1^ showed a decrease by 35%, 42% and 22% in twitch amplitude, contraction time and half relaxation time respectively. *edl* treated with 5 mg kg^−1^ showed an increase of 23% in twitch amplitude without modification of the other parameters. These changes were associated with modification of myofibrillar protein content in all treated groups. Myosin heavy chain isoforms were altered in both skeletal muscles treated with 1 mg kg^−1^.

**Conclusion:**

Exposure to chlorpyrifos can alter the locomotion and produce physiological changes in a dose and muscle type related manner.

## Highlights

• Prolonged exposure to chlorpyrifos induced locomotor impairments.
• Chlorpyrifos exposure modified the characteristics of twitch contraction of mammalian skeletal muscle fibers.
• Structural and functional alterations are reported as adaptation of the fibers muscle to chlorpyrifos exposure.

## 1 Introduction

Even though lots of governmental regulations are being taken to limit the market growth of chlorpyrifos (CPF) over the next coming years (up to 2022), it remains one of the most common organophosphorus pesticides used worldwide. This forecasted market growth can be referred to its increasing demand in various sectors, low cost and convenience [1]. Widely used in agriculture, it afflicts millions of people working in the occupational settings and causes thousands of death yearly throughout the world [2]. According to the US Environmental Protection Agency (EPA), CPF is moderately persistent. It may bioconcentrate at very low levels in ecological systems and thus can be detected in air, food and water. CPF is stable in soils with reported half-lives ranging between 7 and 120 days. Its persistence in soil depends on the soil properties where it has been shown to decrease in soils with alkaline pH [3]. Concentrations of CPF in different environmental matrix are dependent on the formulation, frequency of application and climate. CPF has been detected in drinking water wells at different concentrations depending on site tested as well as the season of testing [4]. People might be exposed *via* three main routes inhalation, dermal and ingestion. While the first two routes are more of a big concern in the occupational settings, the latter route poses an increase in health risk for the general population through eating food and drinking water contaminated with pesticides residues.

The toxicity of CPF results from its neurological effect on nervous system leading to several long term health impacts. Prenatal subacute exposure to CPF in epidemiological studies of children revealed a significant correlation between CPF exposure and neurodevelopmental disorders such as cognitive deficits, impaired motor function [5] and childhood tremors [6]. Locomotor activity in developing animals following CPF exposure was thoroughly investigated comparing to adolescent animals. 5 weeks old rats exposed on postnatal days 1–4 to CPF (1 mg/kg/day, s.c) showed higher locomotor activity comparing to control group [7]. 7 weeks old rats, subjected to same CPF dose regimen in neonate period, showed also an increase in locomotor activity assessed in the elevated plus maze [8]. On the other hand, a decrease in locomotor activity was reported for 3 weeks old rats when tested in open fields further to their neonatal exposure to CPF (1 mg/kg/day, s.c) [9]. Finally, Neonates mice exposed to a single oral dose of CPF (5 mg/kg/day) induced persistent adult behavior and cognitive impairments at age of 2 and 4 months [10]. Even though locomotion in developing rodents assessed by those studies had contrasting results, but all of them reported neurobehavioral alterations. Workers from the occupational settings, who are repeatedly exposed, suffered from lower performance on memory and coordination [11,12]. Prolonged exposure to CPF may also induce same effects as acute exposure including the delayed symptoms. Organophosphate induced delayed neuropathy, a delayed neurotoxic effect, occurs 1–4 weeks after exposure and characterized by pelvic limb weakness, gait abnormality and loss of coordination in people [13].

Despite the fact that CPF is widely studied on the level of central nervous system and peripheral nervous system, the perception of the risk at the neuromuscular junction, especially as a result of prolonged exposure to CPF *via* the ingestion of contaminated food is still not well covered. Therefore the aim of our study is to investigate whether the prolonged daily exposure of adolescent rats for six weeks to two different doses of CPF may affect 1) the locomotor activity assessed *in vivo* by the beam walking test and the beam balance test and 2) the contractile parameters assessed *in vitro* of two skeletal muscles involved in the locomotion: a typical slow-oxidative muscle, the soleus, and a typical fast-glycolytic muscle, the *extensor digitorum longus (edl*).

## 2 Materials and methods

### 2.1 Animals and chemical

30 male Sprague-Dawley rats (6 weeks old, average body weight 200 g) were housed in a plastic cage covered with a stainless steel grid and maintained on a 12L: 12D illumination cycle and temperature 23 °C. The animals had daily access to standardized portion of food pellets (20 g/day/rat) and tap water *ad libitum*. All animals were acclimated for 7 days prior to any treatments. Animal experimentation was performed in accordance with the guidelines for animal care issued by the institutional review board (IRB) at Beirut Arab University.

CPF (O,O' - diethyl- O- 3,5,6- trichloro- 2-pyridylphosphorothioate) (Sigma –Aldrich chemicals) was dissolved in corn oil at two concentrations 1 and 5 mg kg^−1^.

### 2.2 Treatments

Animals were randomly divided into three groups: control, CPF1 and CPF5. Each group contained 10 rats. Animals in CPF1 and CPF5 treated groups were fed daily a diet prepared from standard food pellet mixed with CPF at 1 and 5 mg kg^−1^ respectively for six consecutive weeks. Doses selected, 1 mg kg^-1^ and 5 mg kg^-1^, were based in previous studies where such dose correspond to the oral No Observable Adverse Effect Level (NOAEL) and lowest-observed adverse effect level (LOAEL) respectively [14]. The control group was given only the standard pellet diet with the vehicle (corn oil). Food consumption per cage was monitored daily to check for any changes in food intake. Rats were also observed for any signs of toxic cholinergic symptoms during the entire experimental period. Body weight of each rat was measured and recorded weekly and at the day of the sacrifice.

### 2.3 Behavioral experiments

The locomotion was assessed before the beginning of the treatment (assigned as week 0) and then on a weekly basis during the six weeks of treatment. All behavioral testing occurred in the light phase of the light/dark cycle. Training over 2 consecutive days had preceded the baseline measurements of the behavioral experiments so the rats adapt to the test and reduce stress.

#### 2.3.1 Beam walking test

One traversing segment was used. It consisted of 1.5 m beam with a flat surface of 20 mm width and resting 100 cm above the floor. A black box was placed at the end of the beam. The starting area was brightly lit to provide a motivational stimulus; while the finish point had few food pellets to attract the rat to it. Four testing trials were performed for each rat. Performance on the beam at each trial was quantified manually by measuring traversing latency and number of hind-limb slips that occur in the process. In each trial, the rat was placed on starting position of the beam and simultaneously timer started until the rat cross the beam readily. When the rat’s front feet cross the box at the other end of the beam, the timer was stopped [15].

#### 2.3.2 Beam balance test

Rats were assessed for their ability to balance on a narrow wooden beam (diameter of 1 cm), suspended 70 cm above the floor. Two testing trials were performed for each rat. The rats were placed in the center of the beam and timer started recording the latency time, time from when the animal mounted the rod to when it fell from it [16].

### 2.4 Animals sacrifice and muscles dissection

After the treatment period (6 weeks), the rats were euthanized with an intraperitoneal overdose of sodium pentobarbital (1 ml kg^−1^; 200 mg ml^−1^ solution). The skin was removed from both hind limbs; the *edl* and soleus were dissected respectively. *edl* and soleus removed from one hind limb were directly cooled in liquid nitrogen and stored at −80 °C until they were assessed for contractile protein dosage and myosin heavy chain isoform (MHC) expression. *edl* and soleus removed from the other hind limb were directly transferred to mammalian physiological solution (140 mM NaCl, 6 mM KCl, 5 mM HEPES, 3 mM CaCl2 adjusted to pH 7.35) for the study of *edl* and soleus contractility.

### 2.5 Measurements of muscle contractility parameters

After removal of connective tissue, *edl* and soleus muscle fibers were vertically mounted in a test chamber and connected to a force transducer. The preparation was perfused with the physiological solution at 20 ml min^−1^ and stimulated by electrical pulses at 0.1 Hz. The preparation was then stretched until twitch reached its maximal amplitude. After stretching the muscle to optimal length (L_0_), single or repetitive contractions were elicited with supra-maximal electric current pulses of 2 ms duration passed through platinum electrodes positioned near the muscle and recorded using a bridge amplifier and data acquisition system (Digidata 1200, Axon Instruments) controlled by custom-made software. All subsequent measurements were made at L_0_. After completion of the tension measurements, the cross sectional area of the muscle was calculated by dividing its mass by the muscle density (1.056 g cm^-3^) and L_0_. All experiments were performed at controlled room temperature (19–20 °C) [17].

#### 2.5.1 Twitch measurements

After equilibration, single twitches were elicited. For each twitch; the peak twitch tension (Pt) (measurement between baseline and peak force) which was normalized by the cross sectional area and expressed as (g cm^−2^), contraction time (CT) (time to peak force in ms) and half-relaxation time (1/2 RT in ms) (time for force to decrease to one-half of its peak value) were determined and mean values for each strip were calculated.

#### 2.5.2 Tetanic measurements

Tetanic contractions were produced with trains of stimuli at 20 Hz for the time necessary to establish a clear tension plateau. The trains were set to give a steady plateau. The amplitude between the baseline and the stable portion of tetanic plateau were measured and normalized by the cross sectional area.

### 2.6 Biochemical assessment

Soluble and myofibrillar protein dosage: Frozen *edl* and soleus muscles (50 mg) were washed with 5 vol. washing buffer containing 20 mM NaCl, 1 mM EGTA (pH 6.4), and 5 mM PO4. The muscles were homogenized in ice and the mixture was then centrifuged for 5 min at a speed of 12,000 rpm. The supernatant containing soluble proteins was isolated for later protein dosage. Myofibrillar proteins were extracted from the pellet by adding 3 vol. extraction buffer containing 5 mM EGTA, 1 mM dithiothreitol (pH 8.5), and 100 mM sodium pyrophosphate and incubating overnight at 4 °C on gentle shaking. After incubation, the mixture was centrifuged for 15 min at 12,000 rpm. The supernatant containing myofibrillar proteins was isolated for protein dosage. The soluble and myofibrillar protein concentrations were determined spectrophotometrically at 595 nm using Bradford protocol [18].

#### 2.6.1 Separation of myosin heavy chain isoforms

To analyze the content of MHC I, MHC IIa, IIb, IId/x isoforms in the extracts, sodium dodecyl sulfate polyacrylamide gel electrophoresis (SDS-PAGE) was performed [19]. Samples were prepared within a dilution buffer (5% β-MEtOH, 5% SDS, 20% glycerol, 125 mM Tris, pH 6.8, and 0.1% bromophenol blue) and 2 μg of myofibrillar protein was loaded. Gels run at constant voltage (70 V) for 24 h at 4 °C. All gels were stained with Coomassie R250 and scanned using a Canon digital imaging system. The density of bands was estimated using the UN-Scan-IT software and the percentages of the MHC isoforms were determined by the software from the height (peak intensity) and area of the curve, with area boundaries determined manually.

### 2.7 Statistical analysis

The data are expressed as the means ± S.E.M. Normality was tested using Kolmogorov-Smirnov test. Friedman test was used to show if there is a statistically significant difference in performance over time for the beam walking test, the hind limb slips, and the beam balance test. Kruskal-Wallis test was used to study if there is a significant difference between the three groups (Control, CPF1 and CPF5) at each week. A repeated measures analysis of variance (RMANOVA) was used when data is normally distributed. Then a post hoc analysis with Wilcoxon signed-rank tests was used to confirm where the differences occurred between which groups. Multiple comparisons were controlled using the Bonferroni rule where data is normally distributed. Statistical analysis was performed in SPSS software (version 20, SPSS Inc., Chicago, Illinois, U.S.A.). The significance level for the main treatment effects was set at p < 0.05.

## 3 Results

### 3.1 Body weight

**Fig. 1:**
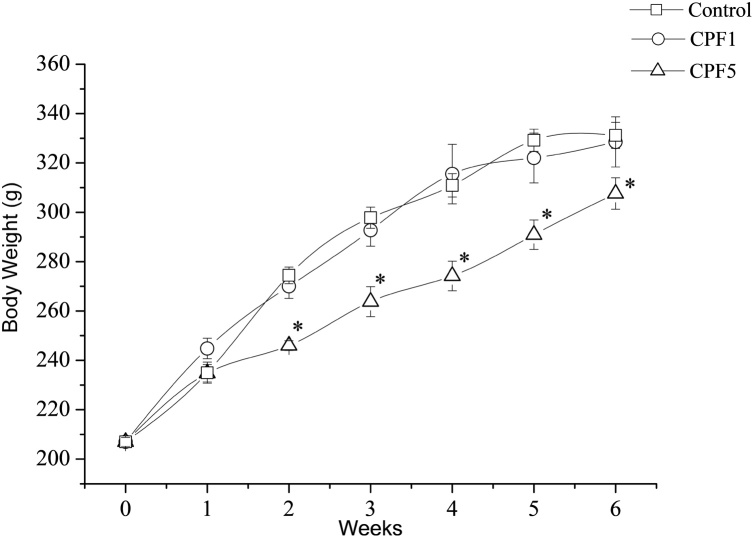
Changes in body weight of the rats within the three groups (n = 10 in each group) over the six weeks after repeated exposure to 1 mg kg^−1^ (CPF1 group) or 5 mg kg^−1^ (CPF5 group) to CPF compared to the control group. Data are shown as mean ± SEM. *: p < 0.05.

During the experimental period, all rats gained weight significantly (p < 0.0005). A significant group-by-time interaction was also observed (*F* (1.895, 3.840) = 3.438, *P* =  0.011). However as shown in Fig. 1, body weight was not affected by the repeated exposure of the dose 1 mg kg^−1^. As for the higher dose, 5 mg kg^−1^, there was a significant decrease in body weight over the 6 weeks (n = 10, p < 0.05).

### 3.2 Behavioral measurements

#### 3.2.1 Beam walking test

**Fig. 2:**
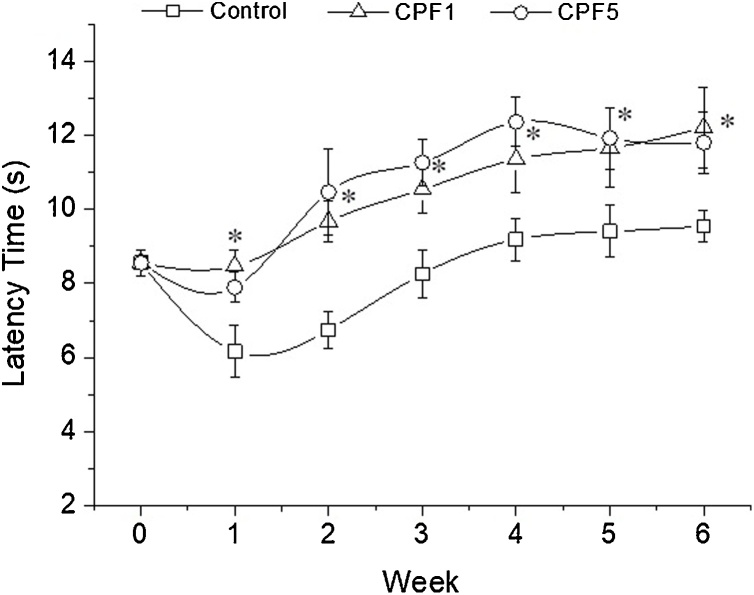
Beam walking test/ latency time. Latency time for rats within the three groups (n = 10 in each group) to cross the beam was recorded on a weekly basis during the 6 weeks of CPF exposure. The data are depicted as mean ± SEM. *: p < 0.05.

Comparison of the repeated measures from the beam walking test using Friedman’s test showed statistically significant difference in performance over time of testing ($\chi^{2}=40.532,\;P<0.0005)$. Further statistical analysis using Kruskal-Wallis test showed a significant difference between the three groups (Control, CPF1 and CPF5). In fact, in control group, there was a decrease in latency time at the first weeks of the study, which is normal since the animals started to habituate to the test. Then a post hoc analysis with Wilcoxon signed-rank tests showed that six weeks of 1 and 5 mg kg^−1^ of CPF exposure have shown a significant effect on locomotor activity assessed by the time needed to cross the beam (Z = -2.158 and Z = -2.391, P = 0.025 and P = 0.013, respectively). As shown in Fig. 2, the increase in latency time was observed starting week 1 by 37% and 28% for CPF1 and CPF5 respectively (Control: 6.16 ± 0.71 s, CPF1 8.47 ± 0.44 s, CPF5 7.89 ± 0.42 s, n = 10, p < 0.05) and persisted over the 6 weeks of the exposure with an increase in time by 28% for CPF1 and 24% for CPF5 comparing to control group (Control: 9.54 ± 0.42 s, CPF1 12.21 ± 1.09 s, CPF5 11.81 ± 0.82 s, p < 0.05). Moreover, no significant difference was observed between CPF1 and CPF5 animals groups during the treatment period.

#### 3.2.2 Hind limb slips test

**Fig. 3:**
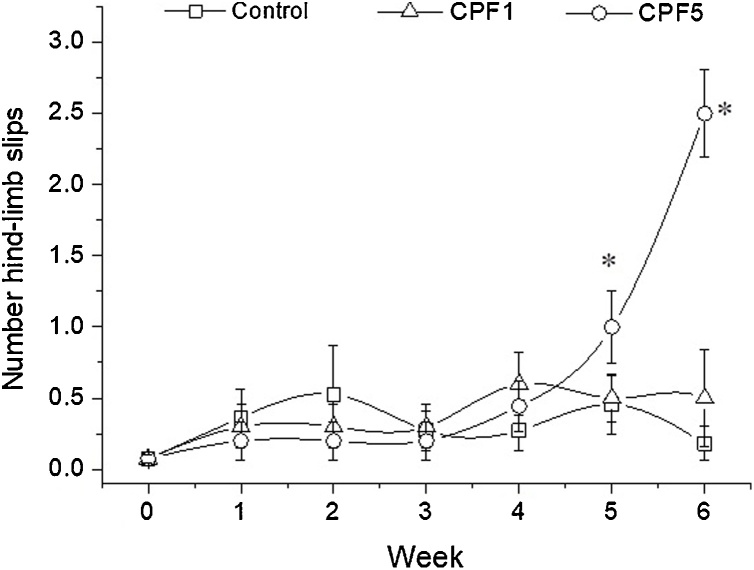
Beam walking test/ hind-limb slips. Number of hind-limb slips for rats within the three groups (n = 10 in each group) was recorded weekly in the beam walking test during the 6 weeks of CPF exposure. The data are depicted as mean ± SEM.*: p < 0.05.

Number of hind limb slips was recorded also from the beam walking test and is presented in Fig. 3. Comparison of the repeated measures from the hind limb slips using Friedman’s test showed significant difference in performance over time of testing ($\chi^{2}=29.146,\;P<0.0005)$. Further statistical analysis using Kruskal-Wallis test and Wilcoxon signed-rank post hoc analysis tests showed a significant difference between the CPF5 and control groups at week 5 (P = 0.002) and week 6 (P < 0.0005). A significant increasing in the mean number of hindlimb slips in CPF5 group *vs* control group was reported (week 5: Control 0.45 ± 0.21, CPF5 1.00 ± 0.25, P = 0.005; week 6: Control 0.18 ± 0.12, CPF5 2.50 ± 0.31, P = 0.0045). No significant difference was observed between CPF1 and control groups during the 6 weeks of experimentation.

#### 3.2.3 Beam balance test

**Fig. 4:**
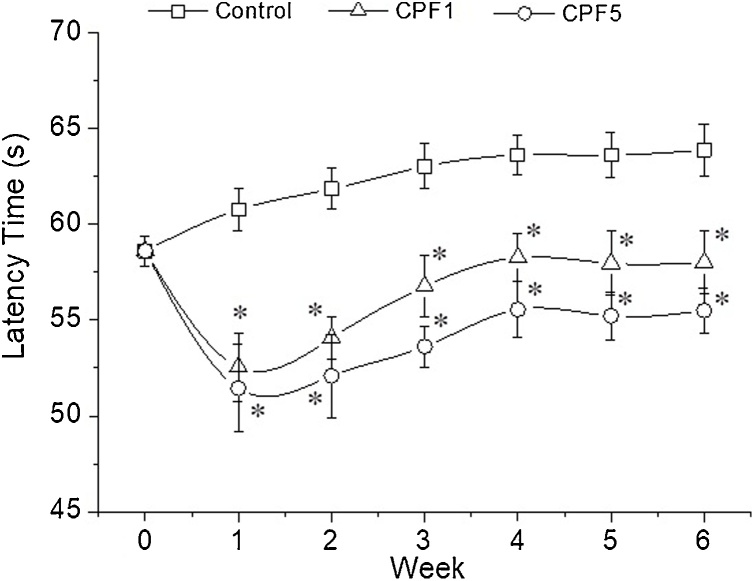
Beam balance test. Latency from when the animal grasped the horizontal rod to when it fell from it was recorded on a weekly basis during the 6 weeks of CPF exposure within the three tested groups (n = 10 for each group). The data are depicted as mean ± SEM. *: p < 0.05.

To further assess the impairments seen in locomotion following CPF exposure, the beam balance test has been conducted. Comparison of the repeated measures from the beam balance test using Friedman’s test showed significant difference in performance over time of testing ($\chi^{2}=42.213,\;P<0.0005)$. Further statistical analysis using Kruskal-Wallis test showed that, one week of exposure to 1 and 5 mg kg^−1^ of CPF induces a significant decrease in latency time by 13% and 15% respectively (Control: 60.76 ± 1.09 s, CPF1: 52.55 ± 1.13 s, CPF5: 51.45 ± 2.29 s, n = 10, p < 0.05). This decrease in latency time persisted up to week six of treatment period by 9% and 13% in CPF1 and CPF5 respectively (Control: 63.88 ± 1.35 s, CPF1: 58.02 ± 1.62 s, CPF5: 55.51 ± 1.16 s, p < 0.05). No significant difference was observed between CPF1 and CPF5 groups (Fig. 4).

### 3.3 Effect of CPF treatment on muscles contractility properties

#### 3.3.1 Effect of CPF treatment on twitch tension

**Fig. 5:**
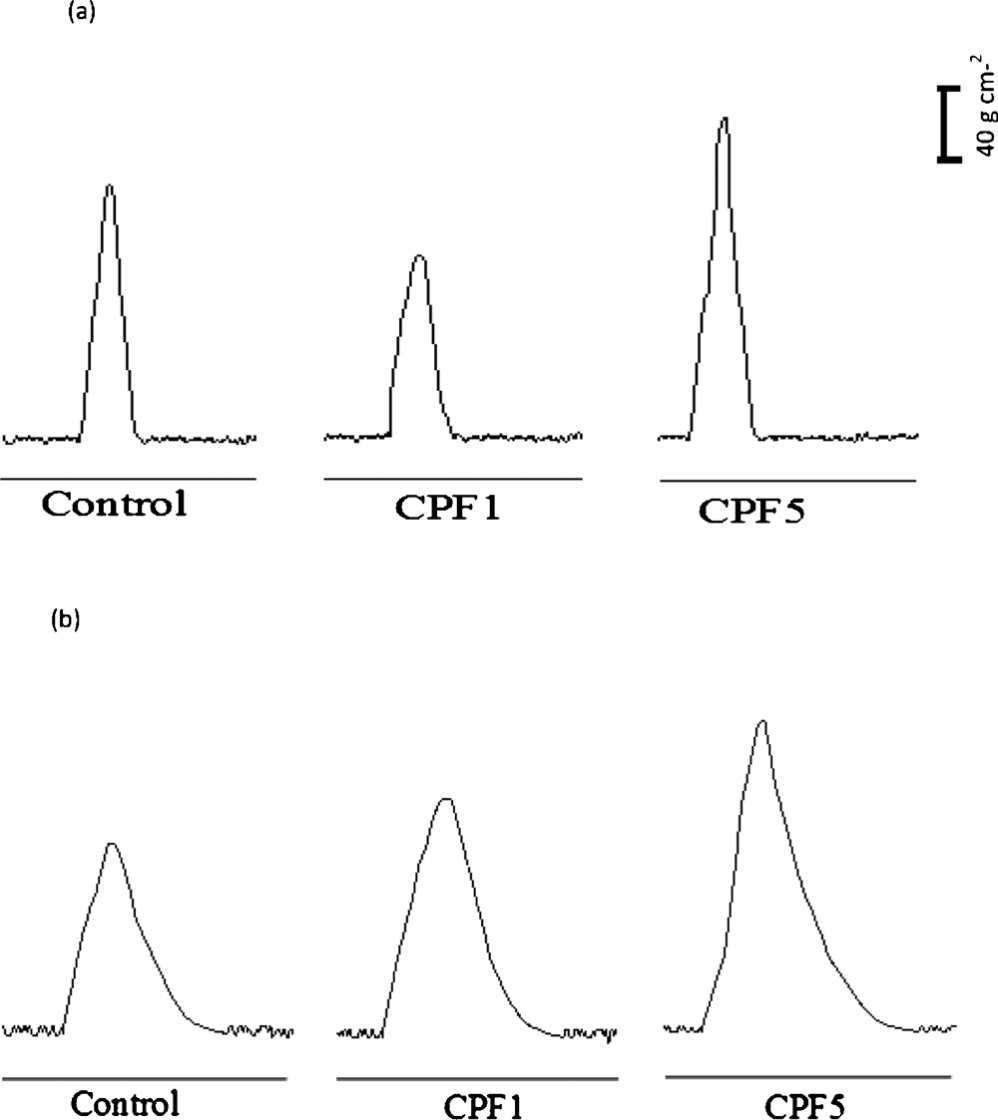
Representative twitch contractions *in vitro*. Responses developed by (a) *edl* fast-twitch fiber preparation and (b) soleus slow- twitch fiber preparation in control, CPF1 and CPF5 after 6 weeks of treatment (n = 10 for each group).

*edl* and soleus contractile properties were recorded the day of the sacrifice (week 6) for the 3 studied groups. Results obtained are shown in Fig. 5.

In control *edl* muscle, twitch tension was characterized by an amplitude of 150.38 ± 7.96 g cm-^2^, contraction time of 54.27 ± 3.49 ms and half relaxation time of 33.06 ± 1.47 ms (n = 10). Treatment with CPF at 1 mg kg^−1^ was shown to induce a significant decrease by 35%, 42% and 22% in the amplitude, contraction time and half relaxation time respectively (n = 10, p < 0.05). However treatment with CPF at 5 mg kg^−1^ was shown to induce a significant increase in the amplitude by 23% (n = 10, p < 0.05) with no significant effect in the contraction time (41.51 ± 5.21 ms) and half relaxation time (29.28 ± 2.75 ms).

As for control soleus muscle, twitch tension was characterized by an amplitude of 106.09 ± 4.72 g cm-^2^, contraction time of 88.23 ± 8.22 ms and half relaxation time of 99.70 ± 5.72 ms (n = 10). As for exposed groups, our results showed treatment with CPF at 1 and 5 mg kg^−1^ induced a significant increase by 25% and 63% in the amplitude of generated twitch respectively (n = 10, p < 0.05) without any significant modification in the contraction time (CPF1: 75.11 ± 8.66 ms; CPF5: 81.25 ± 9.06 ms) and half relaxation time (CPF1: 86.17 ± 6.83 ms, CPF5: 88.23 ± 8.16 ms).

#### 3.3.2 Effect of CPF treatment on tetanus tension

In control *edl* muscle, tetanus contractions were characterized by amplitude of 306.23 ± 29.87 g cm-^2^. Treatment with CPF at 1 and 5 mg kg^−1^ was shown to induce a significant increase by 7% and 27% respectively (n = 10, p < 0.05).

**Fig. 6:**
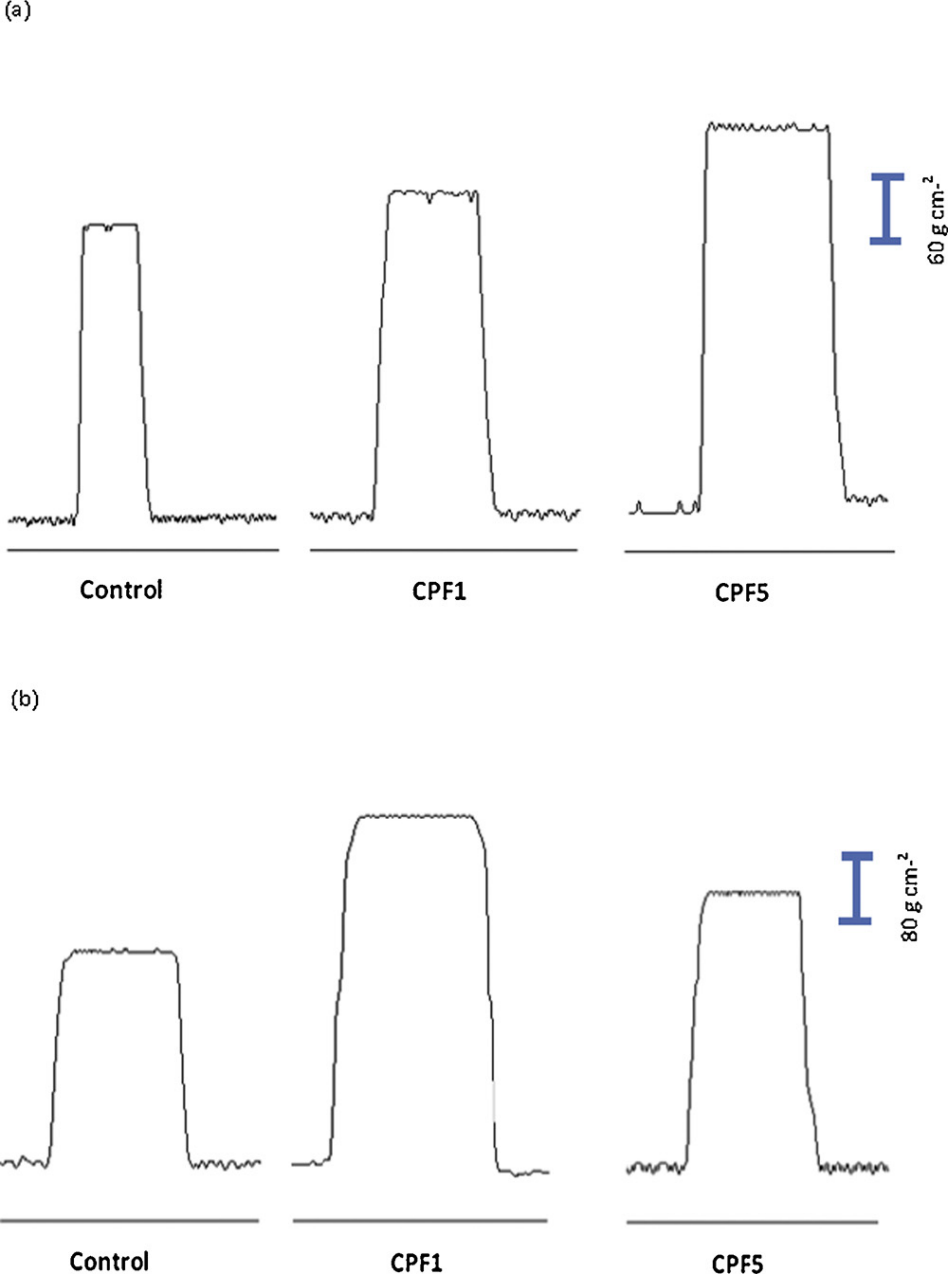
Representative tetanic contractions *in vitro*. Responses developed by (a) *edl* fast-twitch fiber preparation and (b) soleus slow- twitch fiber preparation in control, CPF1 and CPF5 after 6 weeks of treatment (n = 10 for each group).

As for control soleus muscle, tetanus contractions were characterized by amplitude of 260.37 ± 5.55 g cm-^2^. Both CPF1 and CPF5 treatment resulted in significant increase in amplitude of tetanus contractions by 49% and 30% respectively (n = 10, p < 0.05) (Fig. 6).

#### 3.3.3 Effect of CPF treatment on contractile protein contents

**Fig. 7:**
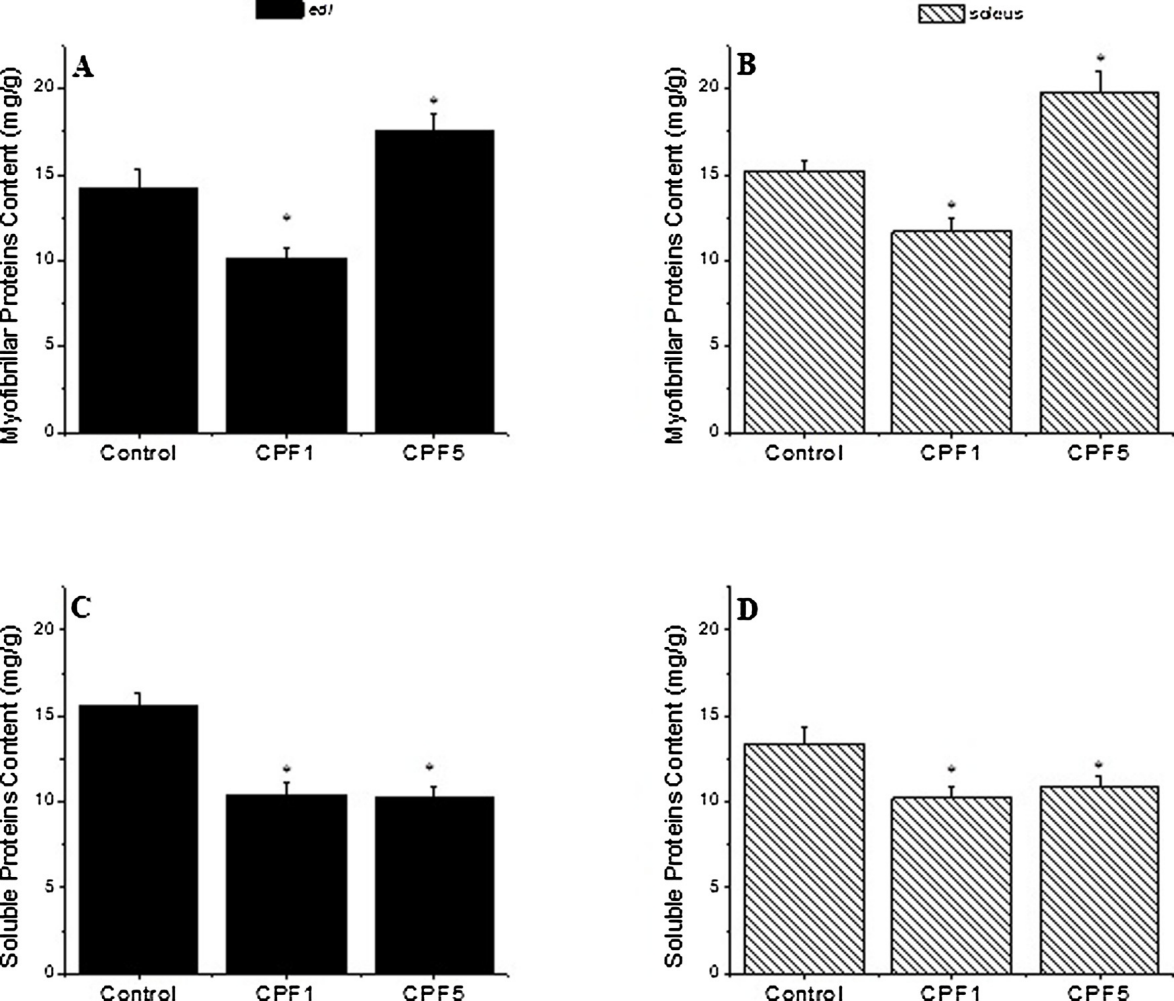
Changes in the myofibrillar protein content in (a) *edl* and (b) soleus and in the soluble total contractile protein content in (c) *edl* and (d) soleus after 6 weeks of CPF exposure. The data are depicted as mean ± SEM. *: p < 0.05.

It has been observed that CPF influences the myofibrillar proteins content, in a dose dependent manner, in both skeletal muscle studied *edl* and soleus. A decrease by 29% and an increase by 23% have been reported in *edl* muscle exposed to CPF1 and CPF5 respectively (p < 0.05). In soleus muscle, a decrease by 23% and an increase by 30% have been determined in CPF1and CPF5 respectively (p < 0.05). The dosage of total contractile protein content of both skeletal muscles studied showed a decrease, dose independent, after 6 weeks of exposure to CPF when compared to control group (p < 0.05) (Fig. 7).

#### 3.3.4 Effect of CPF treatment on myosin heavy chain expression

Control *edl* contained all four MHC types but predominantly types IIa (48.67%) and IIx (42.15%). Electrophoretic analyses of MHC isoform in *edl* revealed a shift toward faster isoform type IIa in CPF1 group. The percent of type IIa fibers was found to increase significantly by 20% compared to control group, whereas the percent of type IIx decreased significantly by 29% (n = 10, p < 0.05). No changes were found for type IIb and type I. Treatment with the 5 mg kg^−1^ dose showed no significant change compared to control group (n = 10).

**Fig. 8:**
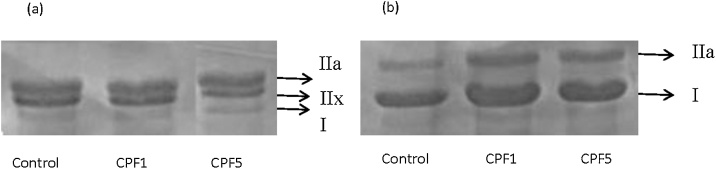
Representative electrophoretogram of MHC isoforms in (a) *edl* and (b) soleus for the three tested groups: control, CPF1 and CPF5.

Control soleus contained only MHC type I (70.15%) and IIa (29.85%) with a predominance of type I. The soleus muscle showed a significant increase in type I by 16% and a significant decrease by 37% in type II fibers in CPF1 group (n = 10, p < 0.05). No effect was recorded for CPF5 compared to control group (n = 10) (Table 1, Fig. 8)

**Table 1:** Effects of CPF1 and CPF5 on Myosin Heavy Chain (MHC) percent and type composition of rat skeletal muscles, *edl* and soleus.

		% Type I	% Type IIa	% Type IIx	% Type IIb
*edl*	Control	3.64 ± 0.91	48.67 ± 2.69	42.15 ± 1.81	5.54 ± 1.02
	CPF 1	2.02 ± 1.31	58.64 ± 3.02*	29.88 ± 1.63*	9.44 ± 2.86
	CPF 5	0.83 ± 0.83	53.62 ± 1.23	35.09 ± 2.35	10.46 ± 0.97
Soleus	Control	70.15 ± 2.46	29.85 ± 2.46	0	0
	CPF 1	81.43 ± 2.84*	18.57 ± 2.85*	0	0
	CPF 5	70.24 ± 1.65	29.76 ± 1.65	0	0

* p < 0.05. The data are depicted as mean ± SEM.

## 4 Discussion

Knowing that agricultural sector relies heavily on the use of CPF, the dietary intake *via* food and water became one of the main routes of exposure [20] and yet not well covered in the preclinical studies comparing to the other routes. Most of the studies emphasize more on acute exposure and to less extent on repeated low level of exposure. The latter is of great importance since it reflects the effect of daily exposure to low level of CPF for the general population. Thus, the present study examined the effect of prolonged dietary exposure of two doses of CPF 1 mg kg^−1^ and 5 mg kg^−1^ in adolescent rats for 6 weeks on the locomotor activity and the physiological parameters of two types of skeletal muscles, a fast twitch skeletal muscle, *edl* and a slow twitch skeletal muscle, soleus, involved in the locomotion.

Overall, and during the 6 weeks of treatment, there were no significant changes in viability of the rats. All of the rats survived the 6 weeks treatments with CPF. Rats did not show any specific sign for cholinergic overstimulation such as salivation, excessive urination, diarrhea, incoordination and fasciculation. The present did not report any changes in food consumption between the treated and the control group. The three groups showed a consistent increase in body weight gain over the six weeks. The body weight was not significantly impaired in animals treated with 1 mg kg^−1^ of CPF. Rats treated with 5 mg kg^−1^ showed less progressive elevation in their body weight gain starting the second week of the exposure and throughout the whole period of the treatment. After 6 weeks of treatment, the mean body weight of CPF5 group was significantly lower compared to CPF1 group and control group. Although an increase in body weight of rats treated with CPF alone or a 13 mixture of chemicals including another type of organophosphate such as methyl parathion has been revealed previously suggesting CPF-induced accelerated differentiation of immature adipocytes into mature fat cells [21,22] and an increase in appetite [23] as a potential mechanism of weight gain, the present however revealed that CPF decreased body weight gain. This finding agreed with some similar previous work reporting CPF-induced body weight loss and where oxidative stress and increased deterioration of lipids and proteins might play a role in this effect observed.

CPF can affect cerebral function in humans thus resulting in an increased occurrence of neurodevelopmental disorders such as cognitive and motor alterations. Gestational or postnatal day exposure to CPF showed a neurotoxic effect in developing organisms manifested mainly as hypolocomotion for preadolescent rats and a hyper locomotion in the adult rats [7,8,10,24,25]. On the other hand, adolescent rats exposed to CPF showed conflicting results in their motor activities level. While some studies reported no alteration in the motor activity [[26], [27], [28]], other studies reported a transient decrease in motor activity due to subchronic dietary exposure [29] and an impairment of motor function manifested in decline in hindlimb grip strength due to subcutaneous injections [30]. Combined exposure to xenobiotics including other organophosphates in long term low dose regiments reflecting the real life risk simulation showed no effect on muscle coordination alongside an increase in locomotor activity of treated rats [31]. Those contradictory results can be elucidated mainly by different route of exposure (subcutaneous injection, intra gastric administration or dietary exposure), varied length of the exposure (one to four weeks), and type of exposure (continuous or intermittent) as well as different methods to measure the animal performance.

Consequently, this study assessed locomotor activity of the rats *via* the beam walking test. Our results have shown that CPF exposure induced locomotor impairments dose independent, which have been clearly manifested as an increase in latency time comparing to control group. The effect was reported starting first week of exposure and persisted all over the 6 weeks of CPF exposure. Moreover, number of hind limb slips measured in the same test showed a significant increase in the mean number of errors in the CPF5 group only at week 5 and 6. Furthermore, to assess the motor function, another test was performed, the beam balance test. Animals treated with CPF have been shown to display deficits in the motor function which exhibited in a significant decrease in the latency time. The performance of the rats showed a significant CPF treatment related effect, dose independent, starting week 1 and till the end of the treatment. To our knowledge, this is one of few studies that addresses impairment in movement and coordination of the hind limbs assessed in adolescent mammalian *via* the beam walking test and the beam balance test further to daily dietary CPF exposure for six consecutive weeks. The motor deficits observed in the behavioral tests can be linked to two types of skeletal muscles in hind limbs involved in the locomotion. During locomotion, *edl* muscle is responsible in large part for the dorsiflexion of the limb which is vital for walking. In rats, the activity of *edl* is initiated when the foot leaves the ground and continues until just after it touches down again. Soleus muscle is known more for its function as weight bearing and stabilization. Its activity in rats begins just prior to foot contact with the ground and continues until immediately before the foot lifts off again [32].

The effect of organophosphate pesticide on skeletal contractile characteristics has been reported previously but mainly for the diaphragm [33,34] and not typically for fast and slow twitch muscles. Our study investigated the findings observed *in vivo* in locomotory activity to the properties observed *in vitro* of the two types of skeletal muscles *edl*, and soleus. Single isolated twitches were firstly measured to provide an insight into the basic physiology by which muscle fibers generate tension. In soleus muscle, we reported a significant increase in peak twitch tension in both CPF1 and CPF5 groups. The result recorded in the present study is consistent with reports that the organophosphate such as CPF and diazinon induced an increase in twitch tension in the diaphragm of the treated rats compared to the non-treated rats [33,34]. The contraction of *edl* from CPF1 group showed a decline in peak twitch tension, contraction time, and half time relaxation. The contraction of *edl* from CPF5 group showed a significant increase in peak of contraction with no significant change in the contraction time and half time relaxation.

Furthermore, tetanic contractions have been measured since most skeletal muscle contractions in the body are of this nature, enabling coordinated body movement and the maintenance of balance. The two doses of CPF treatment resulted in statistically significant increase in tension of tetanus contractions of both skeletal muscles studied.

In skeletal muscle, a direct mechanical interaction between the T-tubule voltage sensor and the sarcoplasmic reticulum (SR) calcium (Ca^2+^) channel/ryanodine receptor (RyR) is specific for excitation-contraction coupling (ECC) mechanism. As response to the depolarization, the voltage sensor changes confirmation and SR Ca^2+^ channel releases calcium into the myoplasm which lead to the activation of the contractile apparatus. The disappearance of Ca^2+^ from the myoplasm is mediated by its final reuptake through the SR Ca^2+^ -transporting ATPase / SERCA receptors leading to muscle relaxation [35]. In this context, the modifications seen in twitch characteristics could have been related to changes at any level of ECC mechanism. In the present study, the amplitude of contractions increased in CPF treated soleus (1 and 5 mg kg^−1^) and CPF treated *edl* (5 mg kg^−1^) muscles might be related to regulation of the expression or the activity of ryanodine receptors leading to an increase of Ca^2+^ released from the intracellular calcium stores needed to trigger contraction, and/or faster Ca^2+^ release. Change in Ca^2+^ sensitivity of contractile proteins is suggested also as a mechanism when there is an increase in muscle contractility. However this might be eliminated since no significant change in time of relaxation was identified. The increase in contractility of the muscle as a result of calcium sensitivity is usually associated with impaired relaxation since calcium will be slowly dissociated from tropomyosin. Even though ECC mechanism is identical in slow and fast twitch muscle, differences in muscle relaxation, calcium binding protein have been determined [36,37].Thus different dose of CPF exposure might not have the same effects on fast and slow twitch muscles. CPF treated *edl* (1 mg kg^−1^) showed a decrease in amplitude, contraction time, and half time relaxation.

Nevertheless, it is possible that the contractile machinery directly contributes to the modifications reported in contractile response of skeletal muscle. Thus myofibrillar protein content and MHC isoforms expression were investigated. Several factors have been studied for their ability to shift MHC expression within skeletal muscle such as training, aging [38,39], and diet [40,41]. Change in MHC expression is considered as an adaptation of skeletal muscle to changes in the pattern of neuromuscular activity.

The decrease in amplitude observed in *edl* exposed to CPF1 could be, in part, associated with a decrease of myofibrillar protein content suggesting proteolysis or inhibition of the synthesis of the contractile proteins. This decrease in myofibrillar was associated with an increase of the MHC type II isoform speculating that the fiber is becoming faster. The decrease of both contraction time and half relaxation time observed can confirm the switching to fast fiber. These results suggest an adaptation of the fibers muscle to changes followed by CPF exposure by making structural and functional alterations.

The increase in amplitude in CPF treated soleus was correlated with a decrease of myofibrillar content in 1 mg kg^−1^ and an increase of MHC I isoform. This increase in force developed, with no changes in the contraction and half-relaxation time, suggest different processes of adaptation of the type muscle due to CPF exposure. This can be explained by the fact that despite soleus is switching to a more MHC type I, there was other functional effects of CPF on the muscle as like alterations in the excitation-contraction coupling mechanism suggested above.

On the other hand, an increase of the myofibrillar proteins content was observed in *edl* and soleus exposed to CPF5. This increase could be correlated with the increase of the force developed. Despite the increase in twitch tension, CPF5 exposure was not associated with changes in MHC isoforms in both types of muscles. These findings are in line with data reported by [42,43] where changes in contractile response in soleus and *edl* following different type of treatment were not associated with changes in MHC isoforms. CPF exerts a concentration-dependent effect causing disruption in the skeletal muscle function on the cellular and subcellular levels in addition to disruptions in neurotransmission [44]. Since pesticides are lipophilic, they can attack the phospholipid bilayer of biological membranes thus causing an alteration of the membrane organization and a disruption in the function and fluidity of the membrane [45]. A chronic co exposure to CPF and deltamethrin resulted in an increase erythrocyte osmotic fragility and lipid peroxidation making erythrocytes highly vulnerable to oxidative hemolysis [46]. Indeed, different types of pesticides such as CPF and fipronil can increase oxidative damage in cells from various organs such as kidney and liver [47,48] as well as in brain and blood tissues [49] leading to disturbances in cell integrity. Aside single stressor studies, it has been shown that a mixture of various chemicals can induce an adaptation to oxidative stress as like increase in catalase activity or other protecting mechanisms depending on dose tested [50]. It is worth mentioning that such kind of stressors combination typically require less of each compound to note the damaging effect seen at higher level when testing each compound separately [51].

In a recent study, it has been reported that regular exercise and use of an antioxidant affected the protein levels of anti-inflammatory and antioxidant markers in soleus muscle [52]. Thus, it will be interesting to examine if the use of anti-oxidant can reverse the oxidative stress induced by CPF in skeletal muscles. In addition, it has been reported that some pesticides induce damage to neurons by generating reactive oxygen species that can affect calcium ATPases carriers [53]. Any variation in the activity or the expression of the Sarcoplasmic reticulum Ca2+ ATPase (SERCA) responsible of calcium homeostasis can be considered harmful to the muscle cell and its function. Thus, further molecular studies are required to determine other mechanisms that might be involved in the modifications observed in our study such as the expression and/or the activity of ryanodine receptors and SERCA.

In summary, our data showed that animals exposed to CPF suffer from impairment in locomotor activity as well as the characteristics of twitch contraction of slow and fast twitch mammalian skeletal muscle fibers. The difference between the contractile responses obtained can be referred to several modifications at the ECC mechanism.

## Funding

This research did not receive any specific grant from funding agencies in the public, commercial, or not-for-profit sectors.

## Transparency document

Transparency document
